# Quality of Life and Social and Psychological Outcomes in Adulthood Following Allogeneic HSCT in Childhood for Inborn Errors of Immunity

**DOI:** 10.1007/s10875-022-01286-6

**Published:** 2022-06-20

**Authors:** Bethany Nicholson, Rupert Goodman, James Day, Austen Worth, Ben Carpenter, Kit Sandford, Emma C. Morris, Siobhan O. Burns, Deborah Ridout, Penny Titman, Mari Campbell

**Affiliations:** 1 Department of Clinical Immunology, Royal Free London NHS Foundation Trust, London, UK; 2 UCL Institute of Immunity & Transplantation, London, UK; 3 Great Ormond Street Hospital for Children NHS Foundation Trust, London, UK; 4 University College London Hospitals NHS Foundation Trust, London, UK; 5 London, UK; 6 UCL Great Ormond Street Institute of Child Health, London, UK

**Keywords:** Very long-term outcome, allogeneic HSCT for IEI, psychosocial outcome, mental health, quality of life

## Abstract

**Background:**

Hematopoietic stem cell transplant (HSCT) is well established as a corrective treatment for many inborn errors of immunity (IEIs) presenting in childhood. Due to improved techniques, more transplants are undertaken and patients are living longer. However, long-term complications can significantly affect future health and quality of life. Previous research has focused on short-term medical outcomes and little is known about health or psychosocial outcomes in adulthood.

**Objective:**

This project aimed to ascertain the long-term social and psychological outcomes for adults who underwent HSCT for IEI during childhood.

**Methods:**

Adult patients, who had all undergone HSCT for IEI during childhood at two specialist immunology services at least 5 years previously, were invited to participate in the study. Questionnaires and practical tasks assessed their current functioning and circumstances. Information was also gathered from medical notes. Data was compared with population norms and a control group of participant-nominated siblings or friends.

**Results:**

Eighty-three patients and 46 matched controls participated in the study. Patients reported significantly better physical health-related quality of life than the general population norm, but significantly worse than matched controls. Patient’s self-reported physical health status and the perceived impact of their physical health on everyday life were worse than matched controls and patients reported higher levels of anxiety and lower mood than the general population. For those where their IEI diagnosis was not associated with a learning disability, cognitive function was generally within the normal range.

**Conclusions:**

Patients who have had a HSCT in childhood report mixed psychosocial outcomes in adulthood. More research is needed to establish screening protocols and targeted interventions to maximize holistic outcomes.

**Clinical Implications:**

Screening for holistic needs and common mental health difficulties should be part of routine follow-up. Information should be provided to patients and families in order to support decision-making regarding progression to transplant and the early identification of any difficulties.

**Supplementary Information:**

The online version contains supplementary material available at 10.1007/s10875-022-01286-6.

## Introduction

### Hematopoietic Stem Cell Transplantation for Inborn Errors of Immunity (IEI)

Inborn errors of immunity (IEI) are rare disorders of the immune system [1] with an estimated prevalence of at least 1 in 15–20,000 [2]. Whilst some IEIs have specific features, including manifestations outside the immune system, an increased susceptibility to infection is their hallmark. Over 400 genes have now been identified to cause specific IEIs, with variability in clinical phenotype and severity [3]. Most IEIs presenting in childhood are of severe phenotype and associated with life-threatening infections. Hematopoietic stem cell transplantation (HSCT) is the treatment of choice and offers the hope of “cure” for severe IEI in childhood. IEI accounts for approximately 20% of the total pediatric HSCT performed in the UK [4] and is limited to three super-specialized centers. An increasing number of children with IEI undergo HSCT each year [5], with more than 75 procedures performed annually in the UK. Due to improvements in donor selection, conditioning regimens and peri-transplant supportive care, over 80% of patients now survive long-term, creating an expanding cohort of adult survivors of HSCT for IEI.

### Medical Outcomes Following HSCT

HSCT for IEI is a high-risk procedure and associated with long-term medical complications, both as a consequence of the underlying IEI and as late effects of the transplant procedure [6]. Factors known to influence medical outcome include genetic IEI diagnosis, donor type, conditioning and engraftment, clinical condition prior to transplant and GVHD [7–11]. In medium-term follow-up studies (around 5 years post HSCT), a number of medical problems have been described, including growth, fertility, hearing, respiratory, gastroenterological and dermatological problems [12–16]. Analysis of later effects specific to IEI is limited but information from studies analyzing HSCT outcomes in other conditions indicates that chronic GVHD, organ dysfunction and secondary malignancies are significant contributors to longer term effects, including late mortality up to 15 years post-HSCT [17, 18].

### Psychosocial Outcomes Following HSCT

Most of the research investigating outcomes following HSCT for IEI has focused on physical outcomes and there has been little assessment of psychosocial measures. Whilst quality of life is referred to [14–16, 19–22], specific standardized measures were generally not used and there was a low threshold for “good” quality of life (e.g., being alive and/or attending school). Seven studies examined psychosocial outcomes and/or quality of life for patients who underwent HSCT for IEI [7, 23–28]. Four of these looked at short-term follow-up in children and only three were limited to patients with IEI. The results indicated some emotional and social difficulties and a long-term impact on quality of life, with variation according to original diagnosis [23, 24, 26, 27]. A fifth study [25] examined late outcome in adult survivors of childhood HSCT, but less than 25% of their sample were patients with IEI (specific number and diagnoses not provided). The final two studies [7, 28] indicate that quality of life is within the normal range for patients who are no longer on immunoglobulin therapy; however, whilst some of their participants were adults, the median age at follow-up was 15 years and the studies used a predominantly child measure to assess quality of life. The pediatric literature shows that there are outcome differences between patients transplanted for IEI and other diagnoses [29] and between patients with different IEIs [27]. We have not identified any studies examining quality of life solely in adult survivors of HSCT for IEI. Within the HSCT population as whole, problems with sleep, energy levels or fatigue, sexual functioning, memory and psychological functioning are reported following transplant [30–34]. It is known, for other populations, that medical late effects and chronic physical health problems can impact quality of life and/or psychosocial outcomes [35–39]. There is also growing evidence to suggest that having a genetic condition in the family also confers an emotional burden [40]. The degree to which these factors impact on outcomes in IEI patients is unknown.

### Aim

The overall aim of the project was to understand the long-term psychosocial outcomes of surviving adults who underwent HSCT for IEI during childhood.

## Methods

### Research Design

The study employed a retrospective cross-sectional stage and a prospective cohort design stage, examining the current functioning of adult patients who underwent HSCT during childhood and comparing it with healthy controls and population norms. Based on earlier data of 22 patients, we observed a mean difference in IQ scores between patient and their sibling controls of 18 units, with sd = 28 [27]. This is equivalent to an effect size of 0.65. However, we anticipate that the effect size is likely to be somewhat smaller for the outcome measures in this study, and an effect size of 0.4 would be the minimum effect size that we would consider clinically meaningful in this group. Ninety-seven pairs would be needed in order to detect an effect size of 0.4 as statistically significant at the 1% level with 90% power; this allows for the assessment of multiple outcomes.

### Participants

All patients aged 16 and over who had undergone an HSCT at Great Ormond Street Hospital (GOSH) or University College London Hospital (UCLH) 5 years or more previously. There were no exclusion criteria.

#### Control Group

The control group was recruited by asking patients to nominate a close friend or sibling. Where multiple matching options were available, patients were first matched for gender, then age. This control group was chosen to control for social factors known to be different in this population compared to other chronic illness groups (e.g., higher rates of Black and minority ethnic individuals).

### Measures

For the patients who had undergone HSCT during childhood, data was collected from current and historical medical notes, through the completion of questionnaires, telephone assessments and a face-to-face psychological assessment. Data was collected in the following areas:

1. Demographic data. Information on age, sex, ethnicity and English as second language was collected from medical notes. Socio-economic status was classified using the simplified National Statistics Socio-economic Classification (NS-SEC; [41]).
2. Psychological and cognitive functioning: The 36-item Short Form Survey (SF-36; [42]) was used as an overall measure of quality of life. Anxiety was measured using the General Anxiety Disorder Assessment (GAD-7; [43]) and depression using the Patient Health Questionnaire (PHQ-9; [44]). Memory, processing speed and attention were assessed using four subtests (digit span, letter-number sequencing, symbol search and information) from the Weschler Adult Intelligence scale – version 4 (WAIS; [45]).
3. Historical and current medical data. Information on diagnosis, age at transplant, conditioning regimen, donor type, and ongoing medical complications (e.g., hearing, skin, fertility, GVHD) and height were gathered from medical notes. The Inventory of Health Status (IHS; [46]) was also used as a measure of general health.
4. Social circumstances. Information on the functional impairment of the patient’s health (including impact on education or employment, leisure activities) was gathered using the Work and Social Adjustment Scale (WSAS; [47]).

For the control group participants, data was collected for overall quality of life, anxiety, depression, cognitive functioning, WSAS, general health, demographic information and social circumstances through completion of the same questionnaires or psychological assessment.

### Procedure

Ethical approval for this study was granted from WALES Research Ethics Committee 6 (Ref: 16/WA/0147). All patients who underwent HSCT at GOSH or UCLH five or more years previously were approached to participate in the research via post, during routine follow-up clinic appointments or through telephone discussion. Control group participants were invited following discussion with the post-HSCT patient. Following provision of consent participants completed questionnaires and underwent a face-to-face psychological assessment. Medical data were collated from medical notes. Patients with learning difficulties, who had low levels of literacy or limited verbal communications skills, were supported by family members or carers to complete as much of the assessments as possible.

### Data Analysis

Patient characteristics were summarized using mean, standard deviation for continuous data or median, interquartile range for skewed data. For categorical data, frequency and proportions are presented. Both patient and control groups were compared with population norms using a one sample *t* test. Comparisons between matched patients and controls were analyzed using a paired *t* test or Wilcoxon matched pairs test as appropriate. Not all patients had a matched control and we compared those with and without a control using a 2-sample test or Mann-Whitney test. Chi-squared tests were used to compare proportions between groups as appropriate. The Kruskal-Wallis and Mann-Whitney tests were used to explore the relationship between levels of anxiety and depression with psychological and physical health factors.

## Results

### Recruitment

Two hundred and thirty-four patients were identified who met eligibility criteria, 81 were found to be deceased and 153 were approached to participate in the study. Eighty-seven of these consented to participate in the study, although four withdrew over the course of the study. Seventy-four patients nominated a healthy control and 48 of these consented to participate in the study, with two later withdrawing. Of the controls, 24 (52%) were siblings (5 (11%) stem cell donors) and 22 (48%) were friends or significant others. None of the siblings was affected by the same IEI.

### *Demographics (Appendix 1)*

Patients and controls were similar in age. The post-HSCT patients had a median age of 23 years (range 16–37). The control group had a median age of 22 years (range 16–41). Less men were recruited into the control group: 57 (69%) of the patients and only 20 (43%) of the controls were male. Patients and controls reported similar ethnicities. Fifty-eight of the patients defined their ethnicity as White British, six as Pakistani, five as other White background, four as Indian, three as Bangladeshi, three as mixed White and Asian, and one as other Asian background and three chose not to provide this data. Thirty of the controls defined their ethnicity as White British, three as Pakistani, three as other White background, three as Indian, one as Bangladeshi, one as mixed White and Asian, and one as other Asian background and four chose not to provide this data. Seventy-two (87%) of the patients and 42 (91%) of controls reported English to be their first language. Using the simplified NS-SEC [41] to categorize social class, more controls reported being in the higher managerial, administrative and professional occupations and less in the intermediate occupations than the patients. Eleven patients (13%) were classified as higher managerial, administrative and professional occupations; 18 (22%) intermediate occupations; 15 (18%) routine and manual; 2 (2%) never worked/long-term unemployed; and 36 (43%) full-time student and one (1%) did not provide data. For the controls, 13 (28%) were classified as higher managerial, administrative and professional occupations; two (4%) intermediate occupations; 11 (24%) routine and manual; no never worked/long-term unemployed; and 19 (41%) full-time student and one (2%) did not provide data. Further breakdown of demographic data of patients with and without matched controls can be found in Appendix 1.

#### Patient Demographics

The median age at transplant was 3 years, with a range from birth (0 months) to 17.8 years. The median time since transplant was 17.1 years, with a range from 5.7 to 37.2 years. Underlying diagnoses were categorized into 3 groups: severe combined immunodeficiency (SCID) (*n*=37); combined immunodeficiency (CID) (*n*=33) and phagocyte disorders (*n*=13). The SCID group included X-linked SCID (*n*=15), adenosine deaminase deficiency (ADA SCID) (*n*=6), purine nucleoside phosphorylase deficiency (PNP SCID) (*n*=1), and all other genetic causes of SCID (*n*=15); the combined immune deficiency (CID) group included Wiskott-Aldrich syndrome (WAS) (*n*=8), CD40 ligand deficiency (*n*=4), X-linked lymphoproliferative disease (XLP) (*n*=3), DOCK8 deficiency (*n*=2), X-linked thrombocytopenia (*n*=1), cartilage hair hypoplasia (CHH) (*n*=1), activated PI3K delta syndrome (APDS2) (*n*=1), CTPS1 (*n*=1), CID secondary to homozygous Rag2 (*n*=1), CARD11 (*n*=1) and genetically undefined CID (*n*=10); and the phagocyte disorder group included CGD (X-linked *n*=5 and autosomal recessive *n*=2), Chediak-Higashi syndrome (*n*=2), leukocyte adhesion deficiency type 1 (LAD1 deficiency) (*n*=2) and undefined neutrophil disorders (*n*=2).

#### Patients with Matched Controls

Demographic details of the patients with and without matched controls can be found in Appendix 1. With the exception of a slightly higher proportion of Black and minority ethnic patients in the group of patients with matched controls and a higher number of students in the group of patients without matched controls, participant demographics were similar across the groups. There was a trend throughout the data for patients with matched controls to report more positive outcomes than for patients without matched controls.

### *Psychosocial Outcomes (Table**1**in ESM)*

#### Quality of Life (SF-36)

Neither the whole patient cohort nor the control group’s scores on the mental component of the SF-36 were significantly different from the general population norm [48] (*p* = 0.32 and *p* = 0.47, respectively) nor was there any significant difference between patients and matched controls (*p* = 0.60). Both patients and matched controls reported significantly better physical health-related quality of life than the general population norm [48] (patients: *p* = 0.046, controls: *p* < 0.01). Patients had significantly worse physical health-related quality of life than matched controls (*p* = 0.02).

#### Anxiety (GAD-7)

Patients reported significantly higher levels of anxiety than the general population norm [49] (*p* < 0.01). There was no significant difference between controls scores and the general population norms (*p* = 0.14). There was no significant difference between patients and matched controls on reported levels of anxiety (*p* = 0.37). Twenty-one patients (25%) had a level of anxiety that would meet IAPT criteria for a clinical diagnosis of anxiety [50].

#### Depression/Low Mood (PHQ-9)

Patients reported significantly lower mood than the general population norm [51] (*p* < 0.01). There was no significant difference between controls and the general population norms (*p* = 0.11), nor between patients and matched controls (*p* = 0.94). Eleven patients (13%) had a level of low mood that would meet IAPT criteria for a clinical diagnosis of depression [49].

#### Cognitive Ability (WAIS-IV; Table 2 in ESM)

Both patients and matched controls group mean scores for all subtests were within the average range (i.e., a scaled score from 8–12). As a number of the conditions are associated with a learning difficulty (e.g., ADA SCID, Chediak-Higashi syndrome and PNP-SCID), further analyses were conducted splitting the patient group into those with a diagnosis in which a learning disability would be expected (LDE, *N* = 9) and those where it would not (no LDE, *N* = 73). Patients with LDE scored significantly worse than the general population on all subscales, except information (digit span *p* = 0.001; letter-number sequencing *p* < 0.01; matrix reasoning *p* < 0.01; symbol search *p* = 0.02; information *p* = 0.67). Patients with no LDE scored significantly worse than the general population on the digit span subscale, but there were no significant differences on the rest of the subscales (digit span *p* < 0.01; letter-number sequencing *p* = 0.12; matrix reasoning *p* = 0.05; symbol search *p* = 0.19; information *p* = 0.54). There were no significant differences between patients with no LDE and the matched controls on any of the subscales (digit span *p* = 0.83; letter-number sequencing *p* = 0.91; matrix reasoning *p* = 0.06; symbol search *p* = 0.13; information *p* = 0.19).

#### General Functioning (WSAS)

Patients reported significantly worse impact of their health on everyday functioning (e.g., work, home and social life) than matched controls (*t*(45) = −2.290, *p* = 0.27).

#### Self-reported Health Status (IHS)

There was no significant difference on self-reported health status between patients and matched controls. However, after excluding one extreme outlier from the data, patients reported significantly worse health status that matched controls (*t*(44) = −2.563, *p* = 0.01).

### Medical Outcomes

Full details of the medical outcomes of this cohort are described in Day et al. [52].

### Relationship between Psychological Health and Physical Health (Table 3 in ESM)

There were no significant relationships found between rates of anxiety and age at transplant, the presence of skin complications, having a short stature, being on IG replacement and current GVHD (*p* = 0.45; *p* = 0.97; *p* = 0.33, *p* = 0.45, *p* = 0.19 respectively). However, there was a trend towards patients who reported having three or more infections in the last year scoring higher on measures of anxiety (*p* = 0.07). Surprisingly, patients who had retained fertility despite transplant conditioning chemotherapy scored higher on measures of anxiety (*p* = 0.06) compared to those diagnosed with infertility. There were no significant relationships in rates of depression and any of the physical health domains exampled (age at transplant *p* = 0.69; 3+ infections *p* = 0.16; skin complications *p* = 0.43; short stature *p* = 0.77; infertility *p* = 0.14; on IG replacement *p* = 0.41; current GVHD *p* = 0.19) as shown in Table 3 in ESM.

## Discussion

### Summary of Findings

The aim of this study was to evaluate the long-term holistic health outcomes of adult patients who underwent HSCT for IEI during childhood. Whilst patients reported significantly better physical health-related quality of life than the general population, they reported significantly worse physical health status and significantly lower physical health-related quality of life than matched controls. Patients also reported significantly more impairment on their work, home and social life due to health reasons than matched controls. The quality of life findings are in line with those of Hamid and colleagues [7, 28] who report that patients, who do not require immunoglobulin replacement therapy, report scores of quality of life comparable to the population norms. A potential explanation for the difference between quality of life ratings and mental health ratings is that expectations of health may impact on quality of life ratings. Individuals, who have experienced very poor health, albeit in childhood, may have different expectations of their health than those who have lead a generally healthy life independent of the degree of impairment their health may have on their daily life.

There were no significant differences between patients and healthy controls or the general population on the mental component of the SF-36. However, patients reported significantly higher levels of anxiety than the general population, and more patients reached criteria indicating clinically significant levels of anxiety than identified in population figures [53] (25% vs 15%) or the untransplanted IEI population [54] (17%). Patients also reported significantly higher levels of depression than the general population. Within the post-HSCT IEI population over double the number of patients scored above criteria indicating clinically significant levels of depression compared to the general population [51] (13% vs 5.6%, respectively). Interestingly, however, this is lower than patients with IEI who have not received a transplant [54] (23.7%).

Patient mean scores for memory, processing speed and attention were within the average range for the population. Patients with LDE scored significantly worse than the general population on all subscales, except information. Patients with no LDE scored significantly worse than the general population on the digit span subscale, but there were no significant differences on the rest of the subscales. The differences in cognitive outcomes are consistent with previous research [27]. Whilst the patients in this cohort are generally performing well there are some differences with the general population that could indicate some slight deficits. Patients performed worse on the digit span test, an assessment of working memory, than controls, but so did the matched control group. This could be indicative of an impact of anxiety on performance, particularly as this was the first test undertaken for both groups. In addition, scores on letter-number sequencing, which also requires memory skills plus the addition of other tasks, were better, again, suggesting that the digit span results may be due to a performance issue.

There did not appear to be any relationship between rates of depression and anxiety and physical health factors known to impact on mental health in other areas, such as the presence of skin complaints or short stature. However, there was a trend in the data for patients who reported having three or more infections in the last year and those who were known *not* to be experiencing infertility to report higher rates of anxiety. Whilst it is important to interpret the results with caution as the numbers are small in each group, the relationship between anxiety and infertility noted here is surprising given the generally recognized relationship between infertility and higher rates of mental health disorders [55] and the role uncertainty is known to play in increasing anxiety. However, it may also be important to consider how the possibility of passing on a genetic condition and/or having a child with a genetic condition can impact on mental health. More research is needed to look at this further. The results may also be a factor of the age and life stage of the cohort with many of the patients being students, not yet in stable relationships and/or possibly not yet contemplating starting a family.

Whilst Day et al. [52] report that younger age at transplant was related to better long-term medical outcomes, age at transplant did not appear to impact on mental health outcomes in adulthood. There is limited literature on the relationship between mental health outcomes and age at transplant; however, in contrast to our results, Nathan and colleagues [56] reported that patients diagnosed with childhood cancer prior to the age of 4 years were a third more likely to experience a severe psychiatric event later in life than children diagnosed after the age of 4. In our study, patients with recurrent infections in the last year of follow-up reported higher rates of anxiety. It may be that patients have learnt to live alongside some of the chronic health conditions they experience, whereas repeated acute infections disrupt life and coping strategies. Further research is required to investigate this so that targeted interventions can be developed to support resilience under different circumstances.

### Clinical Implications

These results suggest that whilst most patients report good quality of life, there are some specific areas of need for adult patients who have undergone HSCT for IEI during childhood. Going forward, whilst it is important to consider medical outcomes and quality of life, difficulties could be missed if routine follow-up does not incorporate psychological screening that includes the specific areas of difficulty identified. Depression and anxiety can impact negatively on psychological, social and employment outcomes which in turn may be costly to the patients themselves, the NHS and wider society [45]. Whilst there are no interventions that have been developed to directly improve quality of life, anxiety and depression could be amenable to change through evidence-based treatments provided by mental health professionals [37, 57].

Further research is needed to investigate outcomes of undertaking regular screening, providing access to timely psychological support if indicated and the impact of providing targeted interventions to those at highest risk of morbidity is important in this cohort. Pediatric and adult services offering HSCT and long-term follow-up could develop resilience-building programs to protect against common difficulties. A good transition process that can pick up on difficulties will be important as transition remains a period of enhanced uncertainty.

It is also important that future policy highlights the need for routine assessment of psychosocial functioning rather than general assessment of patient quality of life. Further research is needed to identify the most important time points for assessment and to identify specific areas of focus. Routine collection of psychosocial data alongside medical data would support this.

There is a trend throughout the data that patients without a nominated matched control participating in the study have poorer scores than those with, suggesting that there may be a correlation between general functioning and peer relationships. Research shows that social isolation is associated with an increased risk for early mortality [58], increased morbidity [59] and poorer mental health [60]. Having a friendship network that provides a good level of support and/or that patients can talk about their concerns with could be a protective factor for patients. Alternatively, the results may be an indication that functioning affects the ability to make such social relationships. This requires further investigation but it may be important to consider interventions that support social integration and lower social isolation.

The results of this study can be used to provide more detailed patient and family information as soon as transplant is discussed. Knowledge of the potential difficulties patients may experience, particularly in relation to their mental health, can allow for earlier identification and intervention.

### Limitations and Strengths

A limitation of this study is the sample was self-selecting and may not be representative of the whole post HSCT population. The cohort is also different from Titman et al. [27] where there were more patients with ADA SCID who showed poorer outcomes and functioning. It may be that the most unwell were not recruited due to poor health and competing demands on time, and the results paint a more positive picture of outcome than is true for all IEI patients post HSCT. Conversely, it is possible the most well were also not recruited, as they no longer attend clinic or have been lost to follow-up, providing more balance to the results.

In addition, not all participants nominated a matched control, and given the trend that patients without matched controls had poorer outcome scores, the generalisability of the comparisons may be affected. In addition, some of the nominated matched controls were siblings. The control group was chosen to control for social factors known to be different in this population compared to other chronic illness groups (e.g., higher rates of black and minority ethnic individuals) and any potential mediating family factors. However, research has suggested that siblings and particularly donor siblings have been shown to report psychosocial difficulties, although some do report positive growth through the experience [61]. Further research is required to investigate any potential differences in outcomes of this group as the numbers in this study are too small to generalize.

Due to the relatively small size of the sample, the study was also unable to control for participant age and/or time since transplant, both of which may affect psychosocial outcomes and should be taken into account going forward. Future research should include a larger, multi-site, international study that can investigate the impact of participant age and time since transplant on holistic long-term outcome for patients who underwent HSCT for IEI during childhood.

Social economic status data (e.g., employment and benefits) was only collected at one time-point (baseline) to avoid complications. This may have affected overall picture of SES, as many were in full-time education at baseline and status may have changed over time.

## Conclusion

Patients who have had a HSCT in childhood for inborn errors of immunity report mixed results in adulthood, with increased impairments in physical health that can impact daily life and higher prevalence of anxiety and depression than the general population. However, patients report good quality of life, and for those where their diagnosis is not associated with a learning disability, cognitive function was generally within the normal range. More research is needed to establish screening protocols and targeted interventions to maximize holistic outcomes.

## Supplementary Information

ESM 1 (DOCX 36 kb)

## Abbreviations

IEI: Inborn errors of immunity
HSCT: Hematopoietic stem cell transplant
GVHD: Graft versus host disease
GOSH: Great Ormond Street Hospital
UCLH: University College Hospital London
NS-SEC: National Statistics Socio-economic Classification
SF-36: 36-item Short Form Survey
GAD-7: General Anxiety Disorder Assessment
PHQ-9: Patient Health Questionnaire
WAIS-4: Weschler Adult Intelligence Scale version 4
IHS: Inventory of Health Status
WSAS: Work and Social Adjustment Scale
SCID: Severe combined immunodeficiency
ADA: Adenosine deaminase deficiency
PNP: Purine nucleoside phosphorylase deficiency
CID: Combined immunodeficiency
WAS: Wiskott-Aldrich syndrome
XLP: X-linked lymphoproliferative disease
CHH: Cartilage hair hypoplasia
APDS2: Activated PI3K delta syndrome
CTPS1: CTP Synthase 1 deficiency
CGD: Chronic granulomatous disorder
LAD1: Leukocyte adhesion deficiency type 1
LDE: Learning disability expected
