# Supplementary material for: Quality of Life and Social and Psychological Outcomes in Adulthood Following Allogeneic HSCT in Childhood for Inborn Errors of Immunity
Source: J Clin Immunol. 2022 Jun 20;42(7):1451–60. doi: 10.1007/s10875-022-01286-6 (PMC9674756; doi:10.1007/s10875-022-01286-6)
Supplement: Supplementary file 1 — (DOCX 36 kb) [file 10875_2022_1286_MOESM1_ESM.docx]

**Supplemental data**

Tables

Table 1: Mean (standard deviation) of psychosocial outcome measures

| **Measure** | **Patients (n=82^[[1]](#footnote-1)^)** | **Comparison between all patients and population norms** | **Patients without matched controls (n=36^[[2]](#footnote-2)^)** | **Patients with matched controls (n=46^[[3]](#footnote-3)^)** | **Comparison of patients with matched controls and matched controls** | **Controls (n=46^[[4]](#footnote-4)^)** | **Comparison between controls and population norms** |
| --- | --- | --- | --- | --- | --- | --- | --- |
| SF-36 Mental  Higher = better QoL | 48.76 (11.16) | p = 0.32 | 47.44 (13.12) | 49.79 (9.39) | p = 0.60 | 48.71 (10.89) | p = 0.47 |
| SF-36 Physical  Higher = better QoL | 52.04 (8.98) | p = 0.046 | 50.53 (9.69) | 53.22 (8.31) | p = 0.02 | 56.79 (6.44) | p < 0.01 |
| GAD-7 score  Median (IQR)  Higher = more anxiety | 4.98 (5.28) | p < 0.01 | 5.21 (6.11) | 4.80 (4.64) | p = 0.37 | 3.98 (4.63) | p = 0.14 |
|  | Mdn = 3.00  IQR = 1.00 – 8.00 |  | Mdn = 2.00  IQR: 0.75 – 11.00 | Mdn = 3.50  IQR = 1.75 – 7.25 |  | Mdn = 2.50  IQR = 0.75 – 6.00 |  |
| PHQ-9 score  Higher = more depression | 4.86 (5.27) | p < 0.01 | 6.06 (6.66) | 3.96 (3.72) | p = 0.94 | 3.89 (4.48) | p = 0.11 |
|  | Mdn = 3.00  IQR = 1.00 – 7.00 |  | Mdn = 3.00  IQR: 1.00 – 8.00 | Mdn = 3.50  IQR = 0.00 – 6.00 |  | Mdn = 3.00  IQR = 1.00 – 6.00 |  |
| WAIS digit span  (N = 45)  Higher = better function | 8.32 (3.61) | N/A | 7.97 (3.73) | 8.47 (3.47) | N/A | 8.93 (2.32) | N/A |
| WAIS letter-number  Higher = better function | 9.00 (3.32) | N/A | 8.50 (3.11) | 9.24 (3.35) | N/A | 9.71 (2.03) | N/A |
| WAIS matrix Higher = better function | 8.80 (3.62) | N/A | 8.39 (3.60) | 9.09 (3.66) | N/A | 10.62 (3.40) | N/A |
| WAIS symbol search  Higher = better function | 9.06 (3.78) | N/A | 8.92 (4.02) | 9.11 (3.63) | N/A | 10.51 (2.46) | N/A |
| WAIS information Higher = better function | 9.88 (3.65) | N/A | 9.72 (3.90) | 9.96 (3.51) | N/A | 11.20 (3.60) | N/A |
| WSAS  Higher = more functional impairment | 7.35 (10.49) | N/A | 8.65 (10.81) | 6.39 (10.26) | p = 0.27 | 2.09 (6.15) | N/A |
| IHS  Higher = worse somatic symptoms | 7.45 (5.79) | N/A | 8.14 (6.00) | 6.91 (5.62) | p = 0.24 | 5.41 (6.92) | N/A |
| IHS (excluding outlier) | n/a | N/A | n/a | 6.91 (5.68) | p = 0.01 | 4.53 (3.55) | N/A |

Table 2: Mean (standard deviation) of WAIS subscales split by whether a learning disability would be expected as part of their condition

| **Measure** | **Patients with LDE (n = 9)** | **Comparison between patients with LDE and population norms** | **Patients with LDE matched controls (n = 6)** | **Patients with no LDE (n = 73)** | **Comparison between patients with no LDE and population norms** | **Patients with no LDE matched controls (n = 40)** | **Comparison between Patients w no LDE & matched controls** |
| --- | --- | --- | --- | --- | --- | --- | --- |
| WAIS digit span | 5.11 (2.80) | p = 0.001 | 5.17 (2.79) | 8.71 (3.51) | p < 0.01 | 9.10 (3.56) | p = 0.83 |
| WAIS letter-number | 5.67 (2.35) | p < 0.01 | 6.00 (1.79) | 9.41 (3.20) | p = 0.12 | 9.90 (3.38) | p = 0.91 |
| WAIS matrix reasoning | 5.44 (3.64) | p < 0.01 | 6.00 (3.90) | 9.22 (3.41) | p = 0.05 | 9.60 (3.40) | p = 0.06 |
| WAIS symbol search | 6.00 (3.94) | p = 0.02 | 6.67 (4.08) | 9.44 (3.61) | p = 0.19 | 9.55 (3.44) | p = 0.13 |
| WAIS information | 6.89 (4.40) | p = 0.67 | 7.00 (3.63) | 10.25 (3.41) | p = 0.54 | 10.45 (3.28) | p = 0.19 |

Table 3: Relationship between psychological and physical health outcomes

|  | **GAD-7**  **Median (IQR)** | **PHQ-9**  **Median (IQR)** |
| --- | --- | --- |
| Age at transplant <1 year (n=30)  1-4 years (n=17)  5 years+ (n=36) | 2.5 (0, 8.5)  5.5 (1, 10)  2.5 (1, 7) | 3.5 (1, 5.5)  5 (1, 8)  3 (0, 7) |
| 3+ Infections No (n=75)  Yes (n=8) | 3 (1, 6.5)  10.5 (3.5, 13.5) | 3 (1, 6)  8 (3, 13) |
| Skin complications No (n=50)  Yes (n=33) | 3 (1, 7)  3 (1, 9) | 3 (0.5, 6.5)  4 (1, 7) |
| Short stature No (n=50)  Yes (n=29) | 3.5 (1, 8)  2 (0, 7) | 4 (1, 8)  3 (0.5, 6.5) |
| Infertility No (n=13)  Yes (n=26)  Infertility unknown (n=44) | 8 (2, 11)  2 (1, 6)  3 (0, 7) | 5 (1, 9)  3 (1, 4)  4 (1, 7) |
| IG replacement No (n=77)  Yes (n=6) | 3 (1, 7)  7 (1, 13) | 3 (1, 7)  4.5 (3, 14) |
| GVHD No (n=73)  Yes (n=10) | 3 (1, 8)  1 (0, 4) | 4 (1, 7)  2 (0, 3) |

**Appendices**

Appendix 1: Group Demographics

|  | **All Patients N = 83** | **Patients without matched control**  **N = 37** | **Pts with matched control N = 46** | **Controls  N = 46** |
| --- | --- | --- | --- | --- |
| **Age (years)** |  |  |  |  |
| Mean (sd) | 23.07 (5.40) | 22.78 (5.44) | 23.30 (5.42) | 22.78 (5.56) |
| Range | 16 - 37 | 16-37 | 16 - 36 | 16 - 41 |
| **Sex (%)** |  |  |  |  |
| Male | 57 (69) | 26 (70) | 31 (67) | 20 (43) |
| Female | 26 (31) | 11 (30) | 15 (33) | 26 (57) |
| **Ethnic background (%)** |  |  |  |  |
| White British | 58 (70) | 27 (73) | 31 (67) | 30 (65) |
| Pakistani | 6 (7) | 2 (5) | 4 (9) | 3 (7) |
| Other white background | 5 (6) | 3 (8) | 2 (4) | 3 (7) |
| Indian | 4 (5) | 1 (3) | 3 (7) | 3 (7) |
| Bangladeshi | 3 (4) | 1 (3) | 2 (4) | 1 (2) |
| Mixed white and Asian | 3 (4) | 1 (3) | 2 (4) | 1 (2) |
| Other Asian background | 1 (1) | 1 (3) | 0 (0) | 1 (2) |
| No data | 3 (4) | 1 (3) | 2 (4) | 4 (8) |
| **First language** |  |  |  |  |
| English | 72 (87) | 31 (84) | 41 (89) | 42 (91) |
| Other | 11 (13) | 6 (16) | 5 (11) | 4 (9) |
| **Social class (Simplified NS-SEC, condensed to 3 ordinal classes, plus FT education)** |  |  |  |  |
| 1. Higher managerial, administrative and professional occupations | 11 (13) | 6 (16) | 5 (11) | 13 (28) |
| 2. Intermediate occupations | 18 (22) | 6 (16) | 12 (26) | 2 (4) |
| 3. Routine and manual occupations | 15 (18) | 5 (14) | 10 (22) | 11 (24) |
| *Never worked and long-term unemployed | 2 (2) | 1 (3) | 1 (2) | 0 (0) |
| Full-time student | 36 (43) | 18 (49) | 18 (39) | 19 (41) |
| No data | 1 (1) | 1 (3) | 0 (0) | 1 (2) |

Appendix 2: Mean (standard deviation) of psychosocial outcome measures by health control category

| **Measure** | **Controls (n=46^[[5]](#footnote-5)^)** | **Non-siblings (N = 22^[[6]](#footnote-6)^)** | **Donor-siblings (N = 5)** | **Non-donor siblings (N = 19)** |
| --- | --- | --- | --- | --- |
| SF-36 Mental  Higher = better QoL | 48.71 (10.89) | 49.36 (9.65) | 39.30 (12.28) | 50.73 (10.95) |
| SF-36 Physical  Higher = better QoL | 56.79 (6.44) | 56.71 (5.75) | 58.26 (3.25) | 56.40 (7.73) |
| GAD-7 score  Median (IQR)  Higher = more anxiety | 3.98 (4.63) | 3.95 (4.32) | 6.20 (4.55) | 3.42 (5.06) |
|  | Mdn = 2.50  IQR = 0.75 – 6.00 | Mdn = 3.00  IQR: 0.75 – 6.25 | Mdn = 5.00  IQR = 2.50 – 10.50 | Mdn = 2.00  IQR = 0.00 – 5.00 |
| PHQ-9 score  Higher = more depression | 3.89 (4.48) | 3.50 (3.49) | 5.60 (3.51) | 3.89 (5.68) |
|  | Mdn = 3.00  IQR = 1.00 – 6.00 | Mdn = 3.00  IQR: 1.75 – 4.50 | Mdn = 6  IQR = 2.50 – 8.50 | Mdn = 1  IQR = 0.00 – 7.00 |
| WAIS digit span  (N = 45)  Higher = better function | 8.93 (2.32) | 8.67 (1.98) | 10.40 (3.51) | 8.84 (2.32) |
| WAIS letter-number  Higher = better function | 9.71 (2.03) | 9.33 (1.85) | 11.00 (3.00) | 9.79 (1.90) |
| WAIS matrix Higher = better function | 10.62 (3.40) | 10.57 (3.44) | 10.80 (2.68) | 10.63 (3.67) |
| WAIS symbol search  Higher = better function | 10.51 (2.46) | 9.76 (2.45) | 11.40 (2.19) | 11.11 (2.42) |
| WAIS information Higher = better function | 11.20 (3.60) | 10.33 (3.94) | 12.6 (3.51) | 11.79 (3.16) |
| WSAS  Higher = more functional impairment | 2.09 (6.15) | 1.14 (2.42) | 5.00 (10.63) | 2.42 (7.68) |
| IHS  Higher = worse somatic symptoms | 5.41 (6.92) | 4.27 (2.21) | 7.20 (3.42) | 6.26 (10.06) |

1. Due to missing data, for the PHQ-9 & IHS excluding outlier n=81 and for the SF-36, GAD-7 & WSAS n=80. [↑](#footnote-ref-1)
2. Due to missing data, for the SF-36, PHQ-9 & IHS excluding outlier n=35 and for the GAD-7 & WSAS n=34. [↑](#footnote-ref-2)
3. Due to missing data, for the SF-36 & WAIS n=45. [↑](#footnote-ref-3)
4. Due to missing data, for the subscales of the WAIS n=45.

   * N.B. For patients who had multiple HSCTs or top-ups, duration was calculated from the patient’s most recent HSCT which involved conditioning. If they have had multiple HSCTs but none of them conditioned, then duration was calculated from their first HSCT. [↑](#footnote-ref-4)
5. Due to missing data, for the subscales of the WAIS n=45. [↑](#footnote-ref-5)
6. Due to missing data, for the subscales of the WAIS n=21. [↑](#footnote-ref-6)
